# Retroaortic closure of thoracic duct in the management of persistent chylothorax: a case report

**DOI:** 10.1186/s13019-019-0917-8

**Published:** 2019-06-04

**Authors:** Francesco Paolo Caronia, Giuseppe Di Miceli, Andrea Macaluso, Damiano Librizzi, Francesco Sgalambro, Alfonso Fiorelli

**Affiliations:** 1 Thoracic Surgery Unit, Ospedale Civico di Palermo, Palermo, Italy; 2 Anesthesiology and Intensive Care Unit, Istituto Oncologico del Mediterraneo, Viagrande, Catania, Italy; 3 Thoracic Surgery Unit, Università della Campania “Luigi Vanvitelli”, Piazza Miraglia, 2, I-80138 Naples, Italy

**Keywords:** Thoracic duct, Chylothorax, Surgical closure

## Abstract

**Background:**

Chylothorax is a life-threatening pathological condition associated with significant morbidity and mortality. If chyle leakage does not close spontaneously with medical therapy, surgical treatment is inevitable. Herein, we reported a case of spontaneous persistent chylothorax from mediastinal seminoma that was successfully closed between the descending thoracic aorta, and the vertebral column through a left mini-thoracotomy.

**Case presentation:**

A 24-year old man with mediastinal seminoma was referred to our attention for management of high output persistent chylothorax (> 800 ml/24 h for 30 days) that did not close with conservative treatment. As the leak was isolated within left upper chest cavity, we planned to close the thoracic duct via Poirier’s triangle by uniportal thoracoscopy. However, the long conservative treatment favoured the formation of multiple, tenacious, and bleeding adhesions that made unfeasible thoracoscopy. A conversion to mini-thoracotomy was performed; by the incision of the posterior parietal pleura, the thoracic duct was isolated and ligated behind the thoracic aorta, in an anatomical space delimited by the 4th and the 5th posterior intercostal arteries and the vertebral column.

**Conclusions:**

Complete resolution of chylothorax was obtained the day after. Patient was discharged on post-operative day 5, and no recurrence was observed during the follow-up.

**Electronic supplementary material:**

The online version of this article (10.1186/s13019-019-0917-8) contains supplementary material, which is available to authorized users.

## Introduction

Chylothorax is a pathological condition characterized by accumulation of lymphatic fluid into the pleural cavity from injury of TD and lymphatic tributaries. It is associated with a significant morbidity and mortality rate as chylothorax leads to malnutrition and immunosuppression through loss of lymphocytes, proteins, fat and respiratory distress [1].

The treatment ranging from conservative to surgery, but the best strategy is still debate as no comparative studies exist. Generally, low output chylothorax (< 1000 mL/24 h) is treated conservatively by low-fat diet, TPN, and chest drainage while surgery is indicated in case of failure. Conversely, patients with high output chylothorax (> 1000 mL/24 h) are candidates for early surgical closure to avoid the deleterious effects of malnutrition and immunosuppression [2]. Mortality rate after early TD surgical closure was reported to be 10%, but it raised to 50% after conservative treatment [3]. TD embolization is a minimally invasive treatment, alternative to surgery, to avoid major trauma in already debilitated patients. Several authors [4, 5] reported a successful rate up to 70%, but these results are difficult to reproduce as TD catheterization and lymphangiography require specific skills available only in selected centres.

Herein, we reported a case of a spontaneous persistent chylothorax from mediastinal seminoma that was successfully closed between the descending thoracic aorta and the vertebral column through a left mini-thoracotomy.

## Case presentation

A 24-year old man with mediastinal seminoma was referred to a local hospital for the management of left pleural effusion. He underwent previous radiotherapy, and was then scheduled for standard BEP (Bleomycin, Etoposide, and Cisplatin) protocol consisted of Cisplatin 20 mg/m^2^ days 1 through 5; Etoposide 100 mg/m^2^ days 1 through 5 for four cycles; Bleomycin 30 mg weekly for 12 weeks (total dose of bleomycin, 360 mg). However, the occurrence of left pleural effusion did not allow to complete the first cycle of treatment. A chest drainage was placed in the left pleural cavity. The fluid had a triglyceride level of 450 mg/dL, and resulted to be positive for chylomicrons. Despite Non-oral feeding (NPO) and TPN were provided for 30 days, output chylothorax > 800 ml/24 h persisted. Chest computed tomography scan confirmed the persistence of left pleural effusion with atelectasis of left lower lobe. Thus, he was referred to our attention for surgical treatment.

**Fig. 1:**
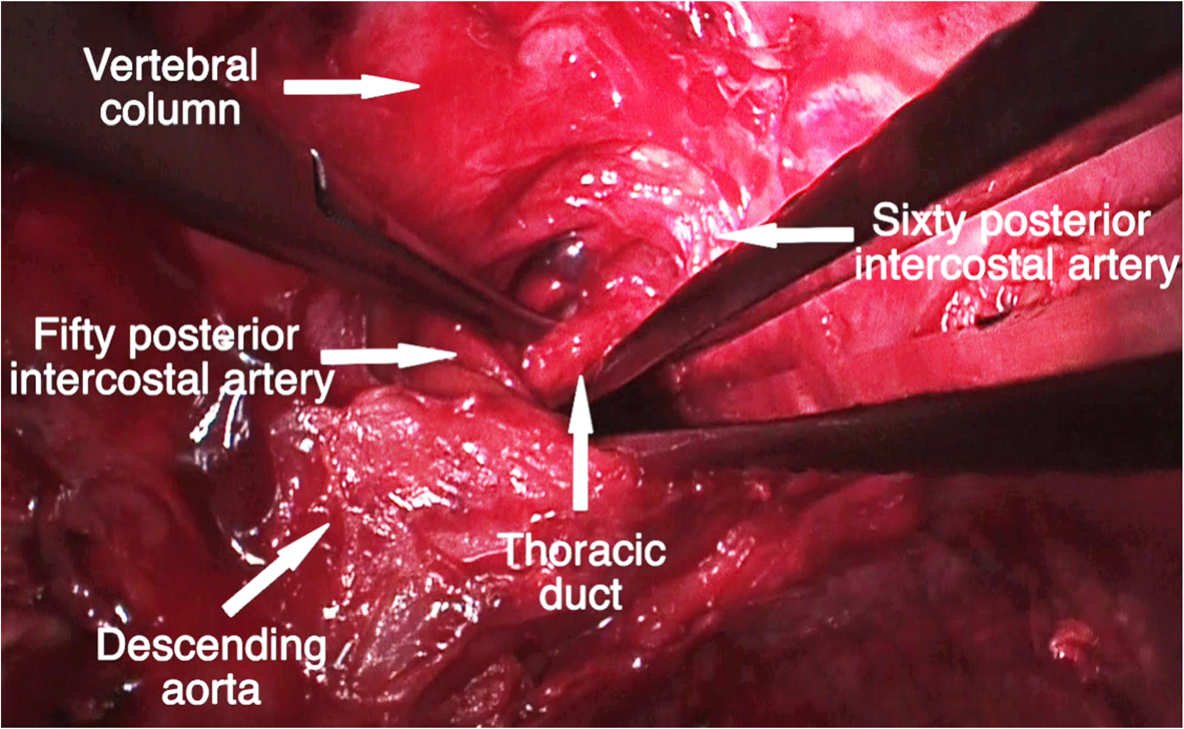
Thoracic duct was identified within anatomical space delimited by descending thoracic aorta, the 5th and the 6th posterior intercostal arteries and the vertebral column

**Fig. 2:**
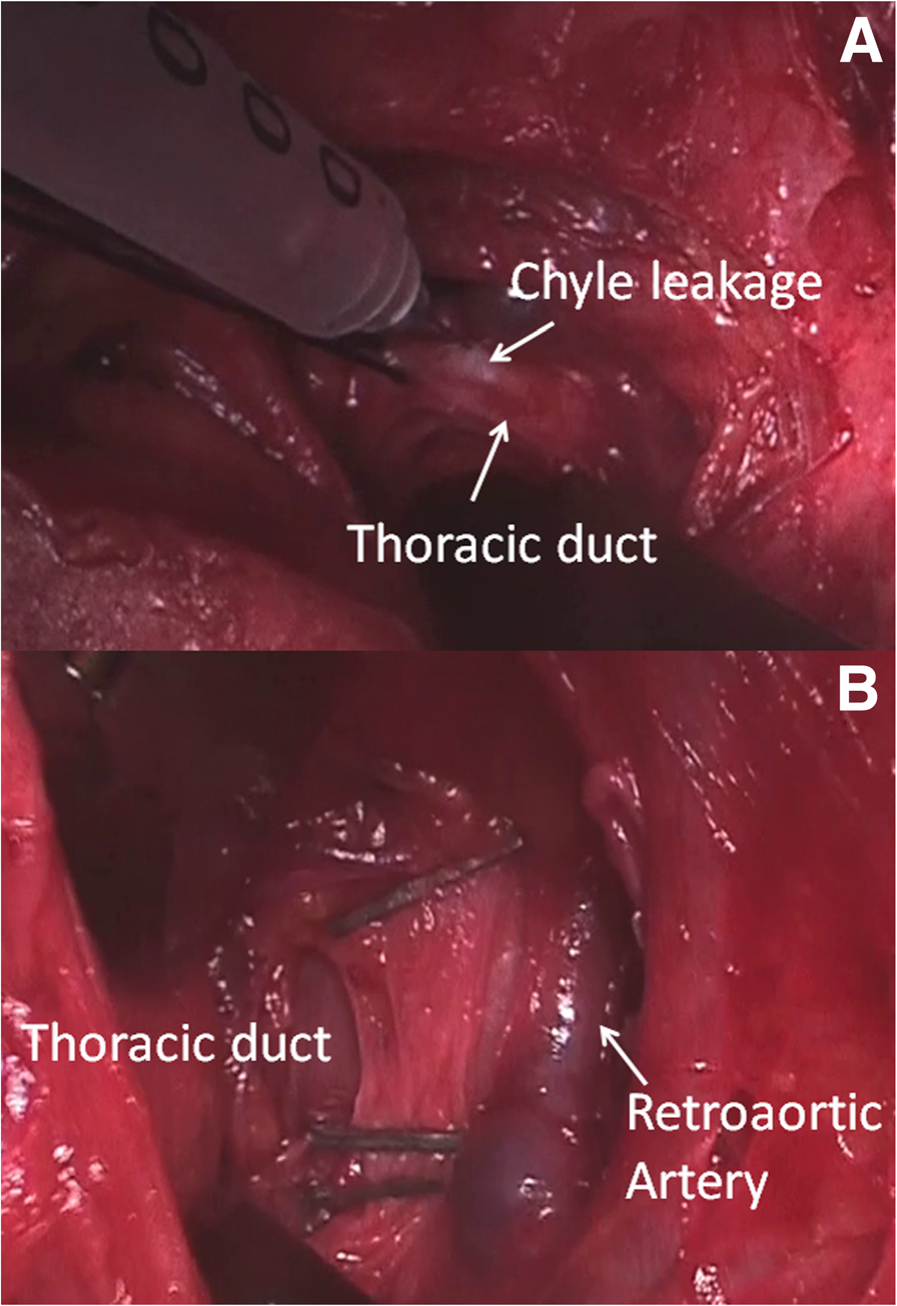
Ability to aspirate chyle using a small needle, confirmed that the isolated structure was the thoracic duct (Part **a**); then, it was clipped at multiple locations along its path (Part **b**)


Under general anaesthesia and selective intubation, the patient was placed in right lateral decubitus. Heavy cream was administered via a nasogastric probe producing a copious white chyle dropping which allowed to identify the leakage. Endoscopic view showed diffuse, tenacious and bleeding pleural adhesions that made difficult the identification of TD at level of Poirier’s triangle and of diaphragm. The uniportal incision was converted to a mini-thoracotomy, and TD was identified near to the esophagus in the anatomical space delimited by descending thoracic aorta, the 4th and the 5th posterior intercostal arteries and the vertebral column (Fig. 1). Ability to aspirate chyle using a small needle, confirmed that the isolated structure was the TD rather than retroaortic artery (Fig. 2/a). Thus, it was clipped at multiple locations along its path (Fig. 2/b). A mechanical debridement of pleural adhesions were performed and associated to pleural lavage with warm povidone-iodine solution diluted with saline. Two chest tubes were left in the chest. The procedure is summarized in Additional file 1: Video S1.

**Additional file 1: ** Video S1. The video edited the main steps of the procedure as the isolation of the thoracic duct and its ligation. (M4V 149001 kb)


Complete resolution of chylothorax was obtained the day after. Patient was discharged on post-operative day 5, and no recurrence of chylothorax was observed during the follow-up. One month later, the patient re-started chemotherapy and completed the four cycles of treatment with complete remission of disease. At 15 months from surgery, the disease is stable without sign of progression.

## Conclusion

Surgery remains the preferred approach for management of persistent chylothorax that does not close spontaneously with medical therapy [1]. Surgical TD ligation may be performed via abdominal, thoracic and cervical approach. Actually, many surgeons prefer to ligate TD using thoracoscopy at the diaphragmatic level as this strategy have the advantage of stopping flow from any accessory tributaries [6]. When TD is difficult to identify, talc pleurodesis alone or associated with surgical decortication may be tried. On the other hand, if the leak is localized in the upper thorax or neck, as in the present, TD is ligated in the Poirier’s triangle, located between carotid artery, aorta arch, and vertebral column [1].

In the present case, since the leak was isolated within left upper chest cavity, we planned to close the TD via Poirier’s triangle by uniportal thoracoscopy, as recently reported by our group [7]. However, the long conservative treatment favoured the formation of multiple, tenacious and bleeding adhesions that made unfeasible thoracoscopy. Thus, a conversion to mini-thoracotomy was performed in order to resect adhesions and to have a better exposure of operative field. Despite all, pleural thickening made difficult the isolation of TD at level of Poirier’s triangle. Other authors [1, 6] in similar cases proposed the mass ligation of all tissues between the aorta, spine, oesophagus, and pericardium above the diaphragm hiatus, via the right pleural space, but also this strategy resulted to be unfeasible due to left access in our cases. Thus, by the incision of the posterior parietal pleural, the TD was isolated behind the thoracic aorta, in an anatomical space delimited by the 4th and the 5th posterior intercostal arteries and the vertebral column. This strategy was planned considering that TD generally travels from the right to the left chest at T3-T5 dorsal between the esophagus and the aorta. In addition, before ligating it, we introduced a needle in the TD and the aspiration of chyle confirmed the exact isolation of this structure.

Our case shows the needing of an early aggressive treatment in patients with persistent chylothorax to prevent the loss of chyle and the formations of tenacious pleural adhesions that could complicate TD ligation through minimally invasive procedures. From a technical point of view, TD ligation behind the descending aorta could be an additional option for physicians when TD is difficult to isolate in the standard anatomical position as at level of diaphragm or in the Poirier’s triangle.

## Abbreviation

TD: Thoracic Duct
TPN: Total Parenteral Nutrition
